# Photodynamic Therapy and Dietary Antioxidants: A Dual Strategy for Genome Stability and DNA Damage Repair

**DOI:** 10.1002/cam4.71032

**Published:** 2025-07-25

**Authors:** J. P. Jose Merlin, Sheeja S. Rajan, Heidi Abrahamse

**Affiliations:** ^1^ Laser Research Centre, Faculty of Health Sciences University of Johannesburg Johannesburg South Africa

**Keywords:** dietary antioxidants, DNA repair, genome stability, photodynamic therapy, reactive oxygen species

## Abstract

**Background:**

Photodynamic therapy (PDT) is an emerging cancer treatment that relies on photosensitizers (PS) activated by specific light wavelengths to produce reactive oxygen species (ROS), effectively targeting malignant cells. However, ROS can also harm surrounding healthy tissues, necessitating strategies to reduce unintended DNA damage. Recent attention has turned to dietary antioxidants as potential agents to protect genome integrity and enhance DNA repair mechanisms during PDT.

**Recent Advances:**

This review explores the complementary roles of PDT and dietary antioxidants in modulating oxidative stress and DNA repair pathways. Key DNA repair systems such as base excision repair (BER), nucleotide excision repair (NER), mismatch repair (MMR), homologous recombination (HR), and non‐homologous end joining (NHEJ) are discussed in the context of their response to PDT‐induced damage. The regulatory role of dietary compounds such as vitamins, polyphenols, flavonoids, phenolic acids, and alkaloids are also examined. Evidence suggests that specific dietary antioxidants can reduce ROS‐induced genomic instability by enhancing the efficiency of DNA repair pathways and modulating gene expression related to repair mechanisms. The combination of PDT with antioxidant intake might reduce mutation risk in healthy cells while preserving the cellular toxicity on cancerous tissue.

**Conclusion:**

Integrating dietary antioxidants with PDT offers a promising dual strategy maximizing tumor destruction while protecting normal cells through enhanced genome maintenance. Continued investigation is necessary to improve this synergistic approach and develop targeted protocols for clinical application, with the aim of enhancing therapeutic outcomes and patient safety.

## Graphical abstract

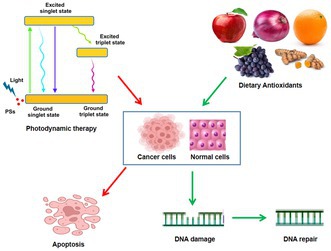

**Abbreviations**
ALKBH2: alkylation repair homolog 2
ATM: ataxia telangiectasia mutated
ATR: ataxia telangiectasia Rad3‐related protein
BER: base excision repair
CAT: catalase
DNA‐PK: DNA‐dependent protein kinase
GG‐NER: global genome NER
GPX: glutathione peroxidase
HR: homologous recombination
Ku70/Ku80: Ku subunits
Lig4: ligase IV
MMR: mismatch repair
MN: micronuclei
MRN: MRE‐11‐RAD50‐NBS1
MTH1: MutT Homolog 1
NER: nucleotide excision repair
NHEJ: nonhomologous end joining
NNKOAc: NNK acetate
NSCLC: nonsmall‐cell lung cancer
PCNA: proliferating cell nuclear antigen
PDT: photodynamic therapy
POLB: DNA polymerase β
POLD: DNA polymerases δ
POLE: DNA polymerases ε
PS: photosensitizer
ROS: reactive oxygen species
RPA: replication protein A
SOD: superoxide dismutase
ssDNA: single‐strand DNA
TC‐NER: transcription‐coupled NER
XLF: XRCC4‐like factor
XRCC4: X‐ray cross complementation group 4 protein

## 1 Introduction

Cancer is anticipated to be the main contributor to mortality globally in 2023 with 10 million deaths and 20 million new cases [1, 2]. The integrity of the genome is the fundamental to cellular function and organismal health continuously posed with difficulties by both internal and external resources of DNA damage [3]. Reactive oxygen species (ROS) is produced while cells go through their regular metabolism under stress which, pose a significant threat to genomic stability [4]. To counteract this threat, cells have developed complex systems for repairing DNA, collectively referred to as the DNA repair pathway, which ensure the faithful maintenance of the genetic code [5]. However, the balance between numerous variables can interfere with DNA damage and repair, including dietary habits [6]. Dietary antioxidants, abundant in fruits, vegetables, nuts, and seeds, have garnered considerable attention for their potential role in preserving genomic stability [7, 8, 9]. Antioxidants counteract the deleterious effects of ROS by neutralizing free radicals and scavenging reactive species before they can inflict damage on cellular components, including DNA [10]. Mounting evidence suggests that the consumption of antioxidant‐rich diets may confer protection against oxidative DNA damage or regulate the activity of enzymes that repair DNA [11]. Central to the genome‐stabilizing function of dietary antioxidants is their ability to interact with essential DNA repair pathway factors [12]. Different processes for DNA repair, including as “base excision repair (BER), nucleotide excision repair (NER), mismatch repair (MMR), homologous recombination (HR), and nonhomologous end joining (NHEJ)”, are coordinated by a complex structure of proteins and enzymes [13]. Research has indicated that specific antioxidants can enhance the efficacy of methods for DNA repair via their impact on expression and activity of these repair mechanisms [14]. For example, polyphenols like quercetin and resveratrol have been shown to upregulate the way in which BER and NER‐related genes are expressed, thereby facilitating the removal of oxidative DNA lesions [15].

Moreover, dietary antioxidants exhibit synergistic effects with endogenous antioxidant defensive mechanisms, which comprise enzymes like “glutathione peroxidase (GPX), catalase (CAT), and superoxide dismutase (SOD)” [16]. Dietary antioxidants can influence redox homeostasis, which in turn may modulate the activity of DNA repair pathways; however, some redox‐dependent modifications are also known to enhance the function of specific DNA repair proteins [4]. Furthermore, some micronutrients, like zinc, selenium, and the vitamins C and E, serve as cofactors or modulators of DNA repair enzymes, augmenting their catalytic activity and enhancing repair efficiency [17]. Understanding the role of dietary antioxidants in genome‐stabilizing DNA repair pathways holds profound implications for human health and disease prevention [18]. Accumulating evidence suggests that inadequate intake of antioxidants or an imbalance between oxidant and antioxidant levels may predispose individuals to increased DNA damage accumulation, genomic instability, and heightened risk of Several medical conditions like cardiac disorders, neurological diseases, and cancers [19]. Conversely, nutrition‐related therapies aimed at boosting antioxidant intake through natural sources or supplements may offer a promising strategy for mitigating DNA damage and promoting overall health [20, 21]. This review examines how dietary antioxidants may interact with DNA repair pathways that help maintain genome stability, focusing on their possible protective effects and relevance to health and disease prevention, while recognizing that detailed mechanisms are not yet fully understood.

Since the late 1990s, the US Food and Drug Administration (FDA) has approved photodynamic therapy (PDT) as a selective cancer treatment [22]. In the presence of oxygen, PDT generates harmful ROS such as singlet oxygen (^1^O_2_), hydroxyl radicals (HO•), and superoxide anions (O_2_•‐) via different photodynamic reactions (PDRs). To commence a PDR, a photosensitizer (PS) must be activated by visible light of a specified wavelength [23]. The light converts the PS electron in the ground state into an excited singlet state. The excited PS can then either return to its ground state and emit fluorescence or change to a triplet state via intersystem crossover [24]. As type I PDR electron transfer occurs, the excited triplet state PS transfers its electrons to the creation of free radical species, which then oxidizes the subcellular substrates. Meanwhile, the excited triplet state PS can transfer energy to oxygen molecules to generate singlet oxygen, and it can trigger cell death by type II PDR energy transfer [25]. It is important to note that both type I and II PDRs are oxygen‐dependent reactions that can occur concurrently depending on PS characteristics and the cellular oxygen level [26]. PDRs have recently been characterized as categories III and IV based on the direct activation of PS. After activation, types III and IV PDRs can immediately exert cytotoxicity, requiring no further processes or oxygen molecules [27]. Under normal conditions, cells maintain a balance of cellular oxidants and antioxidants through redox homeostasis, which ROS control at submicromolar levels. Paradoxically, cancer cells create higher ROS levels as an adaptive strategy, maintaining a persistently prooxidative state that promotes cancer start and growth [28]. However, extremely high ROS levels can cause cancer cell death. As a result, this adaptive mechanism produces antioxidant systems against excess ROS and becomes redox resetting, contributing resistance to many ROS‐mediated cancer therapies, including PDT [29]. PDT improves selectivity by using PS that preferentially accumulate in tumor cells and activating them with localized light to generate ROS that selectively kill cancer cells while minimizing damage to surrounding healthy [2]. This review focuses on the mechanism behind the interaction between PDT and dietary antioxidants, as well as their combined effects on DNA repair and genomic stability.

## 2 Dietary Antioxidants as ROS Scavengers

Dietary antioxidants are essential to maintaining health by scavenging ROS, which are extremely reactive substances created by the body's regular metabolic processes [30]. Radicals that are superoxide anion, hydroxyl radical, nitric oxides, and hydrogen peroxide are examples of ROS that can harm lipids, proteins, and DNA in cells, resulting in oxidative stress and a host of disorders [31]. Peroxidase and ROS oxidize substances containing phenol rings to phenoxyl radicals, which then produce a thyl radical by oxidizing GSH. Consequently, this radical unites with GSH to generate a disulfide radical anion. Through resonance or by giving free radicals more places to attack, the radical may be stabilized by the unsaturated C–C double bond next to the carboxylic acid group [32]. As a prooxidant, ferulic acid increases the generation of ROS in both whole and isolated mitochondria. Research utilizing isolated mitochondria revealed that the interaction of ferulic acid enters the respiratory chain on complex III level is the source of ROS generation [33, 34]. Antioxidants counteract the harmful effects of ROS by donating electrons to neutralize them, thereby defending against oxidative stress. Beta‐carotene, vitamins C, vitamin E, flavonoids, and polyphenols present in whole grains, nuts, fruits, and vegetables are instances of common dietary antioxidants. These antioxidants possess different mechanisms of action and can work synergistically to combat ROS [35]. Vitamin C, for example, acts as a potent water‐soluble antioxidant, scavenging ROS both intra‐ and extracellularly. It also regenerates vitamin E, enhancing its antioxidant activity. Vitamin E, on the other hand, is an antioxidant that is soluble in fat that shields cell membranes from lipid peroxidation [36]. Beta‐carotene, a precursor of vitamin A, quenches singlet oxygen and peroxyl radicals, while flavonoids and polyphenols exhibit diverse antioxidant properties, including metal chelation and enzyme inhibition [37]. Antioxidant‐rich diets have been linked to a lower chance of contracting long‐term illnesses such as heart diseases, cancers, and neurological problems [38]. Studies suggest that antioxidant‐rich foods may help mitigate oxidative stress, inflammation, and cellular damage, thereby promoting overall health and longevity [39, 40]. However, excessive intake of antioxidants through supplements may have adverse effects and disrupt redox balance [41].

## 3 Dietary Antioxidants and P53

Flavonoids, a C15 category of polyphenols, are known to prevent cancer progression and promote DNA repair through p53‐mediated pathways in human cells via antioxidant properties [12]. Plant flavonoids have gained attention in the last two decades as a potential treatment regimen for disease alleviation. Recent studies have shown the capacity of flavonoids to decrease chronic inflammation, specifically via regulating the p53 response [42]. Many flavonoids have identified the p53‐mediated pathway as an appealing target for controlling inflammation and cancer. Flavonoids play a role in regulating transcription factors such as NF‐κB, Nrf2, and AP‐1, which affect inflammation, DNA damage, cell cycle, and apoptosis during tumor cell proliferation. Dietary antioxidants can be used to activate apoptosis in cancer models; we focused on flavonoids and their ability to prevent inflammation and cancer, with an emphasis on p53‐mediated mechanisms [42].

### 3.1 Overview of p53

The p53 protein is necessary to regulate cell division and stop the development of tumors [43]. As a tumor suppressor, it controls the integrity of the cellular genome and trigger several biological responses to avoid stress or DNA damage [44]. Activation of genes linked when DNA damage occurs, cell cycle arrest, DNA repair, and apoptosis occurs due to the action of p53, according to the extent of damage [45]. This ensures that either damaged cells are repaired or destroyed to prevent the propagation of mutations [46]. Because of its crucial function to maintain genomic stability, p53 is frequently recognized to be the “guardian of the genome” [47]. Mutations or dysregulation of p53 are commonly associated with various cancers, highlighting its importance in cancer prevention [48]. Despite its critical role in tumor suppression, p53 is a complex protein subject to intricate regulatory mechanisms, and ongoing research seeks to elucidate its functions in health and disease [49].

### 3.2 Mechanism of p53

**FIGURE 1:**
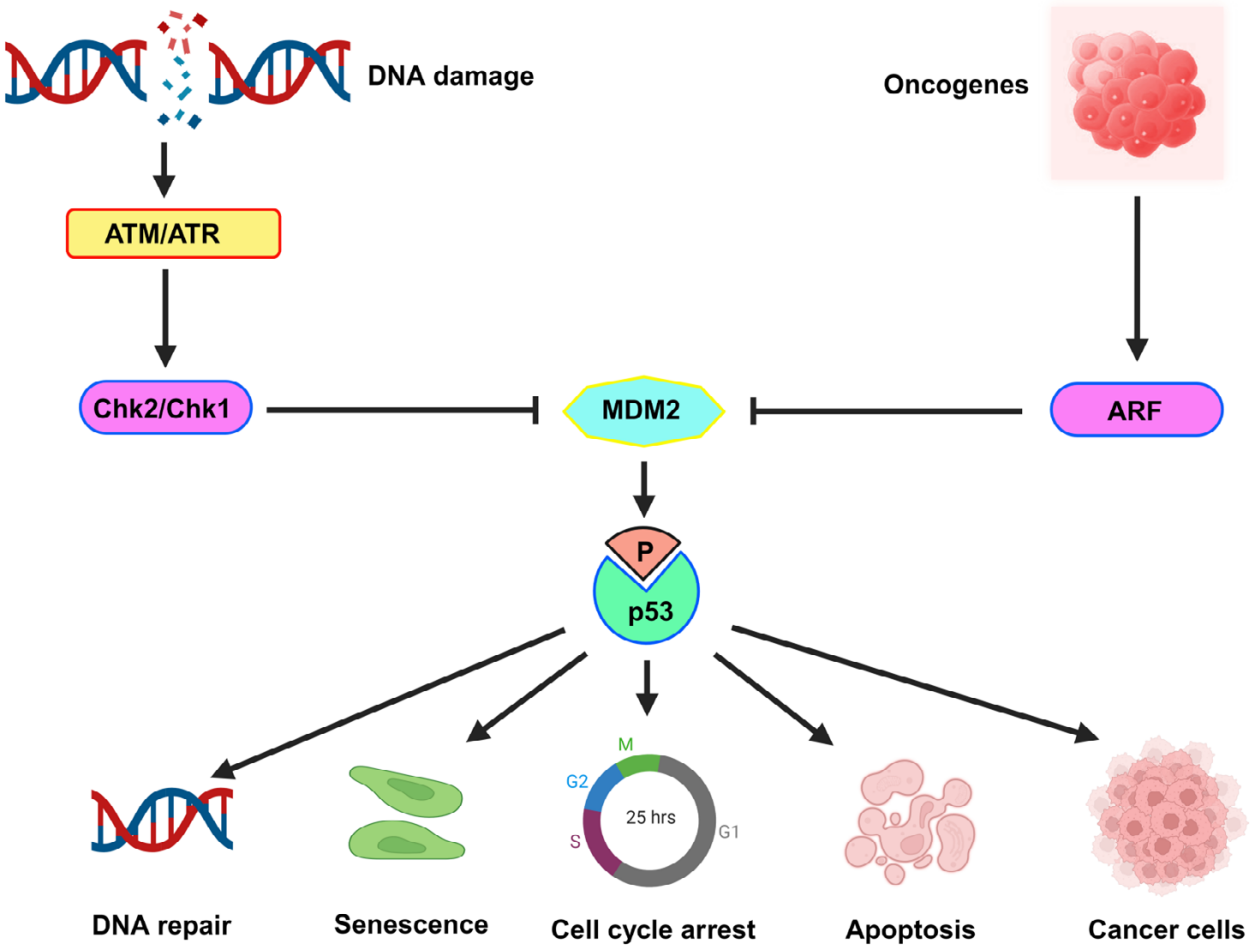
P53 mechanism. p53 is a key tumor suppressor that maintains genomic stability by responding to DNA damage and oncogenic stress. Damage triggers ataxia telangiectasia mutated (ATM) and ataxia telangiectasia and Rad3‐related (ATR) kinases, which activate checkpoint kinase 2 (Chk2) and checkpoint kinase 1 (Chk1), respectively, leading to p53 activation. Oncogenic signals also induce alternate reading frame (ARF), which inhibits mouse double minute 2 homolog (MDM2), a negative regulator of p53, allowing p53 to remain stable. ATM, ATR, and checkpoint kinases phosphorylate p53, leading to its activation, which promotes DNA repair, halts the cell cycle, induces senescence, or triggers apoptosis depending on the severity of the damage. When p53 is lost or mutated, these safeguards fail, enabling cells with DNA damage to proliferate and drive tumor growth.

Under typical cellular circumstances, MDM2 maintains low p53 levels, a proteasome‐mediated E3 ubiquitin ligase that promotes p53 degradation [47]. However, when cells encounter internal or external stressors, posttranslational changes cause p53 to stabilize and become activated. This results in p53 accumulation both in the nucleus and in other cellular compartments [50]. Activated p53, functioning as a transcription factor, attaches itself to specific regions of the genome and prevents many target genes from expressing [44]. p53 is a critical tumor suppressor that maintains genomic stability in response to DNA damage and oncogenic stress. DNA damage activates the kinases ataxia telangiectasia mutated (ATM) and ATR (ATM and Rad3‐related), which in turn activate checkpoint kinase 2 (CHK2) and checkpoint kinase 1 (CHK1), respectively. These checkpoint kinases phosphorylate and stabilize p53, leading to its activation [12]. Oncogenic signals can also activate the alternate reading frame protein (ARF), which inhibits mouse double minute 2 homolog (MDM2), a negative regulator of p53, further contributing to p53 stabilization. Activated p53 promotes DNA repair, induces cell cycle arrest, senescence, or apoptosis, depending on the extent of cellular damage (Figure 1), collectively safeguarding the organism against the emergence of genetically unstable cells that promote cancer [51].

## 4 Dietary Antioxidants Regulate the P53 Signaling Pathway

Various studies, both in vitro and in vivo, have shown that certain natural compounds in our diet can influence the immune system to slow down the development and advancement of cancer [52]. They achieve this by reducing cell growth, deactivating cancer‐causing substances, stopping fresh blood vessel development, prompting cells to arrest their development cycle or self‐destruct, and controlling different communication pathways within cells [53]. The protective and anticancer properties of dietary antioxidants have been observed in multiple types of cells, both healthy and cancerous, as well as in animal studies, often through the regulation of the p53 signaling pathway [12]. Numerous research using normal and cancer cell lines, as well as animal models, have demonstrated the chemopreventive and anticancer effects of dietary antioxidants via the p53 signaling pathway (Table 1).

**TABLE 1:** Dietary antioxidants regulate the p53 signaling pathway.

Dietary antioxidants	Experimental model	Mechanism	Observation	References
**In vitro data**				
Vitamin C	H460 and A549 cells	Increased levels of p53, p21, and Bax and decreased level of Bcl‐2	Decrease cell growth and trigger programmed cell death	[54]
Vitamin B6	HT29, Caco2, LoVo, HEK293T, and HepG2 cells	Decreased levels of p53 and p21	Triggers the p53 pathway, regulating p21 mRNA transcription in various cancer cell lines	[55]
Vitamin E	MCF‐7 cells	Decreased level of p53	Accelerated breast cancer growth in mice by decreasing ROS levels	[56]
N‐acetyl‐cysteine	NSCLC cells	Decreased levels of p53 and p65	Suppresses PDK1 expression via PPARα ligand, impedes NSCLC cell growth	[57]
Vitamin formulation	BEAS‐2B cells	Increased levels of p53, ATR, Chk1, γ‐H2AX, and BRCA1	Facilitate DNA repair via single‐strand break repair and HR	[58]
Quercetin	HeLa Cells	Increased levels of p53, caspase‐3, caspase‐9, and Bax and decreased level of Bcl‐2	Disrupts the potential of the mitochondrial membrane and initiates the intrinsic apoptotic pathway	[59]
Apigenin	HCT‐116 and HT‐29 cells	Increased levels of p53, p21, and NAG‐1	Induced colorectal cancer cell growth arrest	[60]
Kaempferol	HUVECs cells	Increased levels of p53, ATM, Fas, and DR5	Decreased viability of HUVECs cells and reduced DNA fragmentation and damage	[61]
Acacetin	HepG2 cells	Increased levels of p53 and Bax	Decreased AKT activity in liver cancer cell lines and the retinoic acid receptor gamma	[62]
Fisetin	HCT116 cells	Increased levels of p53 and caspase‐3	Suggested as a potential therapeutic approach for treating human colon cancers	[63]
Caffeic acid	HeLa cells	Increased levels of p53 and Bcl‐2	Caused apoptosis to HeLa cells	[64]
Caffeic acid	C6 glioma cells	Increased level of p53 and decreased levels of Bcl‐2 and Bax	Significantly decreased when caffeic acid is pretreated with a particular p38 MAPK inhibitor	[65]
Ellagic acid	CaSki cells	Increased levels of p53, p21, and GAPDH	Suppressed tumor growth and promoting cell death, which supports its role as a chemopreventive agent	[66]
Curcumin	HeLa, SiHa and CaSki cells	Increased levels of p53	Disrupts the connection between p53 and E6AP ubiquitin‐protein ligase, the negative regulator of p53, in the presence of human papillomavirus E6 oncoproteins	[67]
Curcumin	U266B1 cells	Increased levels of p53 and p21	Contributed to cell cycle arrest	[68]
Resveratrol	MCF‐7 cells	Increased levels of p53, p21, and p16	Breast cancer cells exhibit moderate cellular senescence	[69]
Resveratrol	HCT‐116, CO‐115 and SW48 cells	Increased level of p53 and decreased levels of PARP and caspase‐3	Alters p53 by monomethylating it at K372	[70]
Red wine	A549 cells	Increased level of p53	Increased the activation of phosphorylated p53 in human lung cancer cells	[71]
Apple flavonoids	BEAS‐2B cells	Decreased levels of p53, Chk1, and ATR	ATR, Chk1, and p53 phosphorylation levels have significantly decreased.	[72]
Dietary antioxidants formulation	BEAS‐2B cells	Decreased levels of p53 and γ‐H2AX	Reduced p53 and γ‐H2AX phosphorylation in cells subjected to γ‐irradiation	[73]
Caffeine	MOLT‐4 cells	Decreased levels of p53, p21, and Mcl‐1	Interacts with the ATM/p53 signaling pathway in MOLT‐4 cells.	[74]
Caffeine	NHF1 cells	Decreased levels of p53 and p21	Resistant upon exposure to γ‐radiation	[75]
Harmine	MCF‐7 cells	Increased level of p53	Activating the expression of the p53 gene is necessary for induced apoptosis in MCF‐7 cells	[76]
Colchicine	CaSki and HeLa cell lines	Increased level of p53 and decreased levels of Bcl2 and Bax	Caused intrinsic apoptosis, which has antiproliferative effects on HPV 16/18‐positive HeLa cells	[77]
**In vivo data**				
Vitamin D	Mice (1α(OH)ase−/−)	Increased levels of p53 and p16 and decreased level of p21	Increased oxidative stress by suppressing Nrf2 transcription, leading to increased damage to DNA	[78]
α‐Tocopherol, Ascorbic acid & β‐carotene	Wistar rat	Increased levels of p53 and MDM2	Provide protection against oxidative stress	[79]
Fisetin	Wistar rat	Increased level of Bcl‐2 and decreased levels of p53, p21, and Bax	Suggested as a safe and effective therapeutic option for bladder cancer	[80]
Curcumin	Wistar rat	Increased levels of p53 and p21	Suppresses diethylnitrosamine‐induced liver changes, suggesting potential against liver cancer	[81]
**Clinical data**				
Vitamin B9	Human	Increased level of p53 and decreased level of Bcl‐2	Prevents gastric cancer in human	[82]

### 4.1 Vitamins

Vitamin C has been shown in in vitro experiments to decrease cell growth and trigger programmed cell death through increased activity of certain proteins like p53, p21, and Bax while reducing the levels of Bcl‐2 in T‐cell groups. Additionally, Harakeh et al. showed that safe levels of vitamin C boosted the production of p53 [54]. Vitamin B6 triggers the p53 pathway, regulating p21 mRNA transcription in various cancer cell lines like HT29, HepG2, HEK293T, LoVo, and Caco2. Elevated p21 mRNA levels in the mice colon on a vitamin B6‐rich diet compared to those on a deficient diet suggest a potential antitumor effect through p53 activation and p21 mRNA elevation [55]. Earlier research indicated that vitamin D heightened oxidative stress by suppressing Nrf2 transcription, leading to increased damage to DNA and upregulates p16/Rb and p53/p21 activation in 1α(OH)ase−/− mouse model [78]. Vitamin B9 could be crucial in preventing gastric cancer by boosting p53 expression and reducing Bcl‐2 levels in the gastric mucosa following folic acid supplementation in humans [82]. Supplementing water‐soluble vitamin E accelerated cancer growth in mice by decreasing ROS levels and downregulates p53 in MCF‐7 cells [56]. Additionally, N‐acetylcysteine suppresses PDK1 expression via PPARα‐induced activation of p53 and downregulation of p65, offering a new insight into how NAC, combined with a PPARα ligand, impedes lung cancer growth [57]. For prevention against oxidative stress, ascorbic acid, vitamin E, and β‐carotene are helpful, although do not directly affect p53 expression in stressed rats [79]. A recent study found that DNA damage caused by the carcinogen NNK acetate (NKKOAc) activates the ataxia telangiectasia Rad3‐related protein (ATR) pathway, leading to γ‐H2AX phosphorylation and Chk1 activation, which in turn initiates the DNA damage response through proteins such as p53 and BRCA1. When cells were treated with a vitamin‐based antioxidant formulation, this response was modulated, suggesting a potential role in limiting oxidative damage and supporting the DNA repair mechanism. This process involves several effector proteins, p53 and BRCA1 to promote single‐strand break repair through the HR pathway [58].

### 4.2 Flavonoids

As per a recent study, quercetin arrests the cell cycle within G2/M phase, which induces apoptosis, hence inhibiting the growth of HeLa cells. This is accomplished by inducing p53, which disrupts the ability of the mitochondrial membrane and triggers the intrinsic apoptotic pathway [59]. Moreover, apigenin triggers the production of p21, p53, and gene‐1 proteins activated by nonsteroidal anti‐inflammatory drugs via kinase pathways consisting of ATM and protein kinase C delta. This activation is crucial for inducing cell cycle arrest in colorectal cancer [60]. Moreover, kaempferol demonstrates potential as an antiangiogenic agent, decreasing the viability of human umbilical vein endothelial cells while reducing DNA fragmentation and damage. This is achieved by upregulating the signaling pathways of caspases 3, 8, and 9, which are mediated by ROS‐induced activation of the p53/ATM pathway. This stimulation leads to increased levels of downstream proteins including DR4, DR5, and Fas/CD95 [61]. Acacetin is an O‐methylated flavone that was discovered in another investigation and effectively hinders tumor development and results with a reduction of cancers in mice. This effect is associated with elevated p53 expression alongside decreased AKT expression and alters retinoic acid receptor gamma in liver cancer cells [62]. Reduction of securin was found to increase fisetin‐triggered apoptosis and reduce the ability of p53‐negative cells to withstand fisetin, suggesting a potential therapeutic approach for treating human colon cancers [63]. Fisetin's ability to prevent bladder cancer has been observed in a rat model of the onset of bladder cancer, where it activates p53 and suppresses the nuclear factor‐kappa B pathway. This suggests fisetin as a safe and effective therapeutic option for bladder cancer [80].

### 4.3 Phenolic Acids

In HeLa cells, caffeic acid increases the levels of p53 protein in a manner that is dose‐dependent and inhibits Bcl‐2 function and causes apoptosis. When this inhibition occurs, cytochrome c is released, and caspase‐3 is then activated [64]. Phosphorylation of p53 at serine 15 is significantly decreased when caffeic acid is pretreated with a particular p38 MAPK inhibitor. However, CAPE impacts ERK activation in C6 glioma cells and may activate other p53 MAPK‐mediated phosphorylation sites [65]. According to a study, ellagic acid inhibits cell division by causing cell death and G1 arrest through p21, thereby suppressing tumor growth and promoting cell death, which supports its role as a chemopreventive agent [66].

### 4.4 Polyphenols

Curcumin elevates p53 levels, facilitating interaction between p53 and quinone oxidoreductase NAD(P)H in cell lines generated from tumors like HeLa, SiHa, and CaSki. Concurrently, this interaction disrupts the connection between p53 and E6AP ubiquitin‐protein ligase, the negative regulator of p53, in the presence of human papillomavirus E6 oncoproteins [67]. Another study proposed that curcumin increased p53 and p21 levels in U266B1 cells to inhibit cell cycle arrest [68]. Moreover, in Wistar rats, curcumin down‐regulates p53 and p21 expression [81]. The p53/p21Cip1/WAF1 pathway is activated by metabolites of resveratrol to cause G2/M phase arrest, which limits cell growth and causes moderate cellular senescence in breast cancer cells [69]. An earlier investigation revealed that SET7/9 mediates the p53 activation caused by resveratrol, indicating that resveratrol alters p53 by monomethylating it at K372 [70]. An additional study evaluated the chemopreventive and anticancer effects of red wine and found that grape‐derived red wine rich in polyphenols may increase the activation of phosphorylated p53 in human lung cancer cells [71]. One investigation observed phosphorylation of effector proteins in BEAS‐2B cells treated with NNKOAc, including p53, Chk1, and Chk2. p53, Chk1, and ATR phosphorylation in cells exposed to NNKOAc were significantly reduced when apple flavonoids were pretreated [72]. A recent study found that pretreatment with AOX2, ascorbic acid, quercetin, or curcumin significantly decreased phosphorylation of γ‐H2AX and p53 in BEAS‐2B cells subjected with γ‐irradiation and NNKOAc treatment [73].

### 4.5 Alkaloids

In human, MOLT‐4 cells and caffeine interfere with the ATM/p53 signaling pathway by reducing p21 overexpression and preventing p53 phosphorylation at Ser15 and Ser392. This action appears to be ATM‐independent, hinting at another cellular target for caffeine [74]. Furthermore, in their investigation of human fibroblasts, Kaufmann et al. noted that caffeine‐resistant upon exposure to γ‐radiation, the phosphorylation of p53 is dependent on ATM [75]. Moreover, breast cancer cells undergo time‐dependent apoptosis when exposed to harmine, with real‐time PCR results indicating that its apoptotic induction in MCF‐7 cells relies on activating p53 gene expression [76] Colchicine inhibits HPV E6/E7, causing p53‐dependent intrinsic apoptosis, which has antiproliferative effects on human cervical cancer cell lines positive for HPV 16/18 in vitro [77].

Some studies have reported adverse or variable effects, such as vitamin E potentially promoting tumor progression by suppressing p53 activity, and curcumin showing inconsistent influence on p53 expression depending on the experimental setting [67, 68, 79, 81]. Additionally, compounds like caffeine have been found to disrupt p53 phosphorylation, indicating that their effects may vary depending on cellular context. Including these findings offers a more balanced and critical perspective on the literature, highlighting the complex interactions between dietary antioxidants, DNA damage response pathways, and p53 regulation [74, 75]. This broader view enhances our understanding of both the promising therapeutic roles and the possible limitations of these compounds in maintaining genomic integrity.

## 5 Genome‐Stabilizing DNA Repair Pathways

One essential mechanism in cells that protects the integrity of genetic material, DNA, is the gene‐stabilizing DNA repair pathways [83]. DNA damage can arise from a variety of internal and external factors. Internally, ROS generated within the cell can lead to DNA damage. Externally, substances including air pollution, xenobiotic exposure, smoking, hypoxia, and ionizing radiation can also induce the production of ROS within cells, contributing to DNA damage [84]. Genomic instability and mutations can result from unrepaired DNA damage, which can ultimately promote conditions like cancer [85]. According to studies, consuming a diet rich in antioxidants either decreases the amount of DNA damage or increases the effectiveness of DNA repair. Furthermore, the stability of the genome depends on dietary antioxidants [86, 87]. Antioxidants found in food have attracted interest as a potential cancer prevention strategy because they can neutralize free radicals that cause DNA damage and may enhance the activity of DNA repair pathways, although they do not directly initiate the repair processes themselves [84]. The genome‐stabilizing DNA repair pathways encompass several distinct repair mechanisms, each specialized in addressing specific DNA damage types [88].

### 5.1 Base Excision Repair Pathway

**FIGURE 2:**
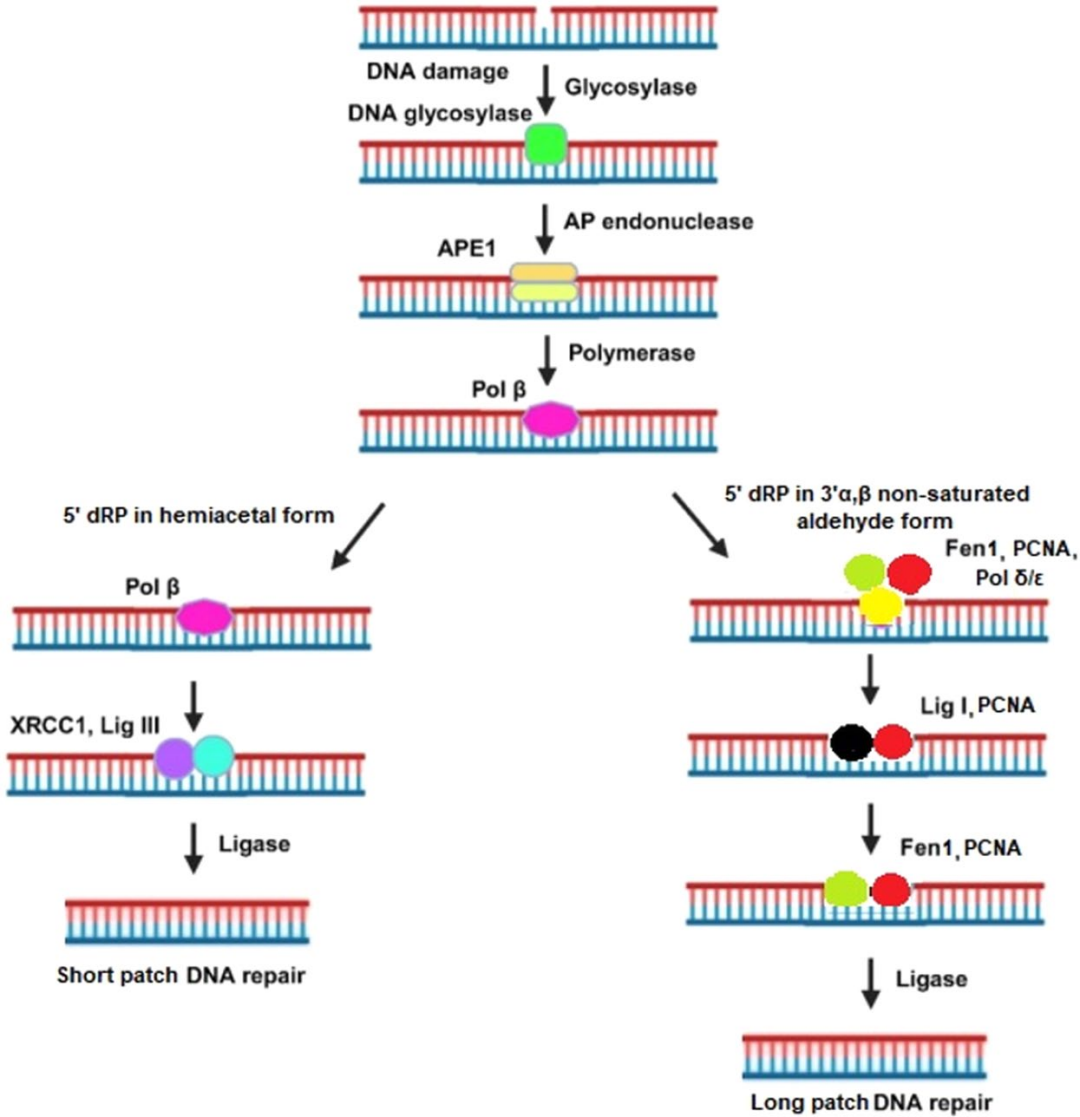
Base excision repair (BER) pathway. Base excision repair is a crucial pathway that repairs oxidative DNA damage using specific protein sets in both the nucleus and mitochondria. It begins when DNA glycosylases recognize and remove damaged bases, creating apurinic/apyrimidinic (AP) sites. These sites are then cleaved by AP endonuclease 1 (APE1), allowing further repair steps. Poly‐ADP‐ribose polymerase 1 (PARP1) binds to the damaged site and helps recruit additional repair proteins. DNA polymerase β fills in the missing nucleotide, and once the repair complex is properly formed, PARP1 dissociates, completing the repair process.

Base excision repair represents the predominant pathway for rectifying oxidative damage to DNA lesions within both the nuclear and mitochondrial compartments, albeit employing distinct protein constituents [89]. The BER pathway is initiated by a lesion‐specific DNA glycosylase that identifies and removes a damaged or inappropriate base, generating an abasic site as the first intermediate in the repair process. While various DNA glycosylases are identified within the nucleus, only a subset is observed within mitochondria, notably comprising uracil‐DNA glycosylase and 8‐oxoguanine DNA glycosylase 1 as principal mitochondrial DNA glycosylases [90]. DNA glycosylases catalyze the cleavage of the N‐glycosidic bond, resulting in the formation of apurinic/apyrimidinic (AP) sites by removing damaged bases from the DNA backbone. DNA glycosylases generate abasic AP sites by catalyzing the hydrolysis of the N‐glycosidic bond between the damaged base and the deoxyribose sugar. This reaction removes the aberrant base and leaves the sugar‐phosphate backbone intact but lacking a nucleobase, thereby creating an abasic site. The original phrasing suggesting a ‘joining’ of lesions with deoxyribose is misleading, as the enzymatic activity involves base excision rather than conjugation. AP endonuclease 1 (APE1) is generated to this location [91]. BER involves a coordinated series of enzymatic steps requiring a DNA glycosylase, AP endonuclease, DNA polymerase, and DNA ligase. The process is initiated by removal of a damaged base by a DNA glycosylase, generating an abasic AP site. AP endonuclease then cleaves the site to produce 3′‐OH and 5′‐dRP termini. DNA polymerase β fills the resulting gap and removes the dRP residue, and DNA ligase seals the final nick to complete repair [92]. Upon DNA damage, PARP1‐mediated ADP‐ribosylation facilitates the recruitment of several key components of the BER pathway, including DNA ligase III, polynucleotide kinase/phosphatase (PNKP), DNA polymerase β (POLB), and X‐ray repair cross‐complementing protein 1 (XRCC1) [93]. DNA polymerase gamma only functions in mitochondria, whereas POLB functions within the nucleus (Figure 2) [94]. Upon assembly of the BER complex, poly‐ADP‐ribose polymerase 1 (PARP1) acquires sufficient negative charges, leading to its dissociation from the DNA lesion, facilitating effective repair by the BER complex [95].

DNA damage has been observed in nonsmall cell lung cancer cells following resveratrol treatment, and this effect is enhanced when combined with the antifolate drug pemetrexed [96]. Although curcumin has been reported to offer protective effects against DNA damage in lymphocytes exposed to long‐term arsenic, some studies have shown that it downregulates the expression of OGG1—an essential glycosylase responsible for initiating BER of oxidative DNA lesions. This suggests that curcumin's role in BER‐related DNA repair may be more complex than previously thought and may not universally enhance all components of the BER pathway [97]. A similar reduction in both proteins was noted in breast cancer cells induced by cigarette smoke and treated with resveratrol alone. This treatment also led to an increase in p21 levels, which interfered with the interaction between FEN1 and PCNA, ultimately suppressing the long‐patch BER pathway. Additionally, other essential components of this repair mechanism, including DNA ligase I and polymerases β, δ, and ε, were found to be downregulated [98]. Berberine enhanced the responsiveness of triple‐negative breast cancer cells to chemotherapeutic agents such as cisplatin, camptothecin, and methyl methanesulfonate by weakening XRCC1‐dependent BER, which in turn led to an increase in double‐strand DNA breaks [99]. Honokiol, a biphenolic compound derived from Magnolia officinalis, exhibits strong anticancer properties and demonstrates greater toxicity toward tumor cells compared to normal cells. It has been shown to suppress the function of X family polymerases, specifically polymerases β and λ, thereby disrupting the BER pathway and enhancing the vulnerability of various cancer cells to treatments like bleomycin and temozolomide [100].

### 5.2 Nucleotide Excision Repair Pathway

**FIGURE 3:**
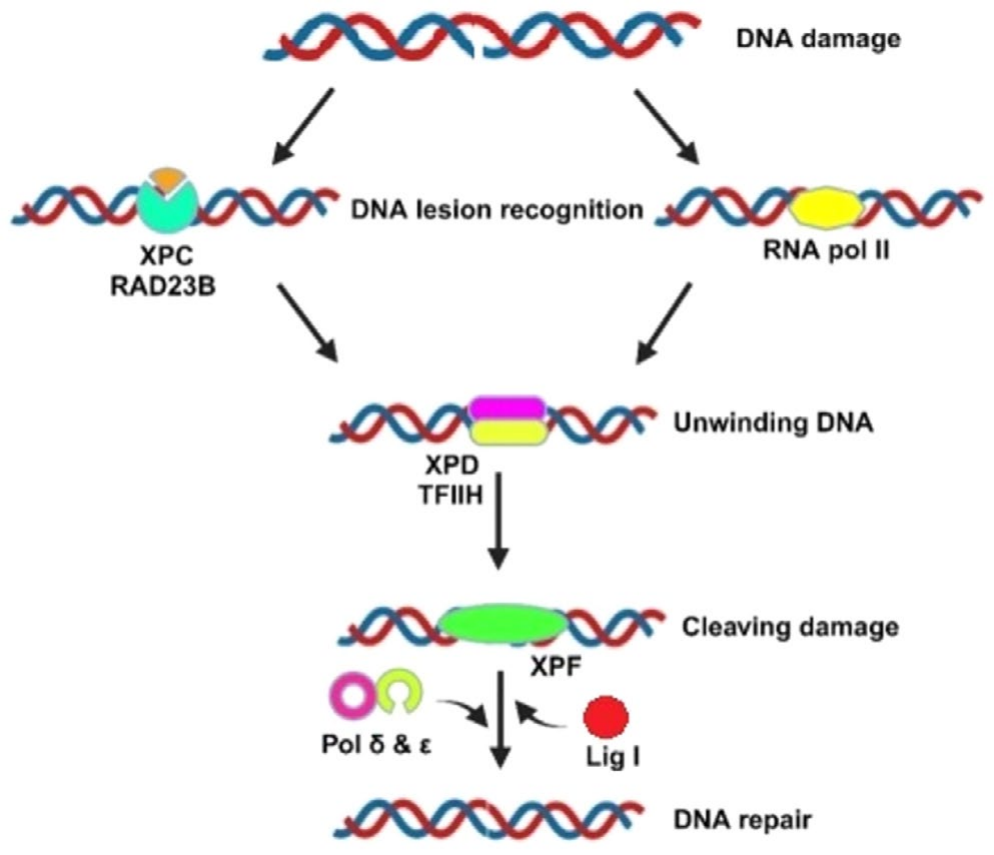
Nucleotide excision repair (NER) pathway. Nucleotide excision repair begins with the identification and removal of bulky DNA lesions. In global genome NER (GG‐NER), the XPC‐RAD23B complex detects the damage throughout the genome. In contrast, transcription‐coupled NER (TC‐NER) is initiated when RNA polymerase II stalls at lesions during transcription. Both pathways converge as repair proteins such as XPD and XPF are recruited to unwind and excise the damaged DNA strand. The gap is then filled by DNA polymerases δ (POLD) or ε (POLE), and the strand is sealed by DNA ligase I to complete the repair process.

Nucleotide excision repair typically involves four main steps: recognition of DNA lesions, unwinding of the DNA helix, making incisions to remove the damaged segment, and subsequent DNA synthesis and ligation, as illustrated in Figure 3. NER employs over thirty proteins to address various types of DNA damage effectively [95]. Global genome NER (GG‐NER) and transcription‐coupled NER (TC‐NER) are two separate sub‐processes that are part of NER. Primarily, these sub‐processes diverge during the first recognition phase. DNA damages are recognized in GG‐NER by the XPC‐RAD23B complex, which is mostly composed of UV excision repair protein radiation sensitive 23 homolog B and xeroderma pigmentosum complementation group C. This role in TC‐NER is performed by a stalled RNA pol II complex, which is necessary for enlisting many enzymes linked to TC‐NER [101]. After the DNA lesion is identified, the two sub‐processes come together because of the recruitment of xeroderma pigmentosum complementation group D, base transcription initiation factor IIH, and DNA strand unwinding, which exposes large lesions. The damaged DNA sequences are then excised by the NER machinery, which is made up of xeroderma pigmentosum complementation group F and DNA excision repair protein 1. DNA polymerases δ and ε (POLD and POLE) then produce new sequences. Finally, any remaining nicks in the DNA strand are sealed by DNA ligase I [102].

Resveratrol exhibits a synergistic cytotoxic effect with etoposide in nonsmall‐cell lung cancer (NSCLC) cells by suppressing XRCC1 expression. Targeting XRCC1 downregulation may offer a promising strategy to enhance etoposide sensitivity and improve therapeutic outcomes in NSCLC patients [103]. When used alongside carboplatin, curcumin helps lessen its harmful side effects by specifically enhancing NER in bone marrow cells. This protective effect is achieved through the upregulation of BRCA1, BRCA2, and excision repair cross‐complementation group 1 (ERCC1) [104]. Triptolide, a bioactive diterpene triepoxide from traditional Chinese medicine, exerts potent antitumor effects by covalently binding to the XPB/ERCC3 subunit of TFIIH, thereby inhibiting its DNA‐dependent ATPase activity. This interaction disrupts RNA Polymerase II–mediated transcription and likely impairs NER, highlighting XPB as a key molecular target and positioning triptolide as a promising tool for transcriptional research and anticancer therapy development [105]. Similarly, in pancreatic cancer cell lines, triptolide acted synergistically with oxaliplatin by downregulating several essential proteins involved in the NER pathway, including XPA, XPB, XPC, ERCC1, XPD, and XPF [106]. The combination of low doses of Minnelide and oxaliplatin shows significant potential as a novel therapeutic strategy for the treatment of pancreatic cancer [106]. Retiegeric acid B enhances the effectiveness of cisplatin in hormone‐refractory prostate cancer cells by modulating NER, specifically targeting proteins such as ERCC1, TFB5, and RPA1, as well as influencing MMR, likely through the involvement of MSH2 and MSH6 proteins [107].

### 5.3 Mismatch Repair Pathway

**FIGURE 4:**
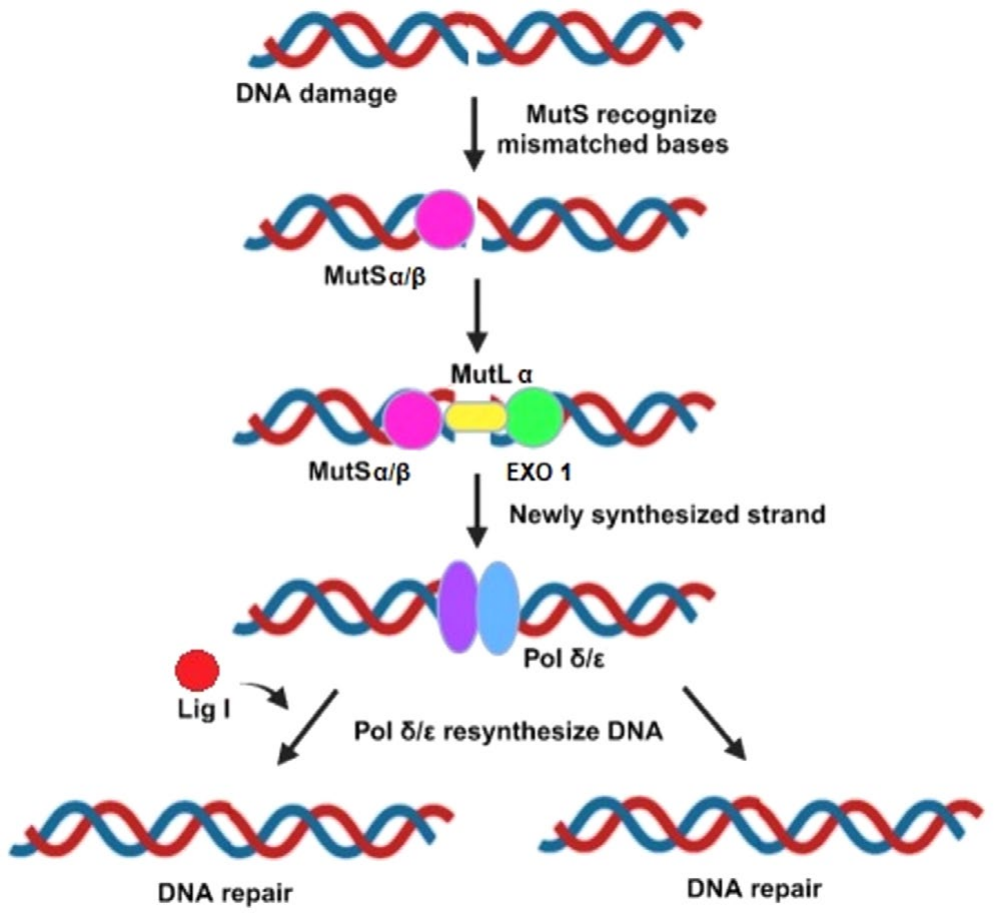
Mismatch repair (MMR) pathway. Mismatch repair is a crucial DNA repair mechanism that fixes errors that occur during DNA replication. The process is initiated when the MutSα complex or MutSβ complex recognizes the mismatch. The MutLα complex is then recruited and activates endonuclease activity to introduce a nick near the mismatch. The repair machinery distinguishes the newly synthesized strand by the presence of nicks or gaps left by the replication process. Exonuclease 1 (EXO1) degrades the DNA segment containing the error, and DNA polymerases δ (POLD) or ε (POLE) resynthesizes the correct sequence, followed by ligation to restore the DNA strand.

Recent research indicates that MMR, in addition to BER and NER, is essential for repairing oxidative DNA damage. However, the precise molecular mechanism underlying MMR's elimination of DNA lesions remains incompletely elucidated. It appears that MMR‐associated proteins are capable of differentiating between parental and newly synthesized strands of DNA. Utilizing the parental DNA strand as a repair template, MMR rectifies DNA lesions present on the newly synthesized strand [108]. MMR comprises two crucial protein complexes: mammalian counterparts of prokaryotic MutS and MutL [109]. In Figure 4, MutS homolog proteins initiate the MMR process by recognizing DNA mismatches. MutL homologs are then recruited to the site, where they facilitate strand incision and the recruitment of exonuclease 1 (EXO1), which removes the error‐containing DNA segment. POLD/POLE then synthesizes the replacement strand using the parental template, aided by proliferating cell nuclear antigen (PCNA), which enhances polymerase processivity. Finally, DNA ligase 1 (LIG1) seals the remaining nick, completing the repair and restoring DNA integrity [95]. Presently, research linking the MMR pathway to heart diseases remains relatively sparse. However, one study identified a potential association between MMR regulation and human heart failure. The study specifically noted decreased levels of human MutY homolog, a human MutSα‐interacting DNA glycosylase connected to the BER pathway that supports MMR repair [110].

Resveratrol has been found to influence various DNA repair pathways in MCF7 breast cancer cells by downregulating the expression of several genes associated with these processes, including those involved in MMR [111]. MMR plays a crucial role in the activity of curcumin, as cells lacking this system, particularly those with defects in MSH2 and MLH1 proteins, exhibit heightened sensitivity to the compound. In contrast, cells with a functional MMR system can activate CHK1 and undergo G2/M phase arrest prior to apoptosis, while deficient cells bypass this checkpoint and proceed directly to apoptosis [112]. Retiegeric acid B enhances the efficacy of cisplatin in hormone‐refractory prostate cancer cells by influencing NER, specifically targeting proteins like ERCC1, TFB5, and RPA1, as well as MMR, likely involving MSH2 and MSH6 proteins [107].

### 5.4 Homologous Recombination Pathway

**FIGURE 5:**
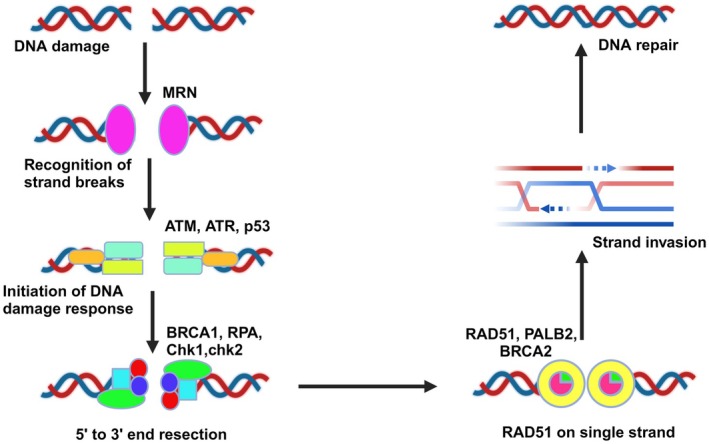
Homologous recombination (HR) pathway. Homologous recombination is a vital repair mechanism for double‐strand breaks (DSBs) in DNA. DSBs are initially detected by key proteins, including p53, ATM, ATR, and the MRN complex, which trigger the repair process. Once identified, end resection is initiated by BRCA1, exposing single‐strand DNA (ssDNA), which is then protected by replication protein A (RPA). This step activates ATR, which further supports the repair process. RAD51, along with BRCA2 and PALB2, plays a crucial role in facilitating the search for homologous sequences and repairing the break using a sister chromatid or homologous chromosome, thereby ensuring genomic stability.

Homologous recombination is crucial for tapping into the genetic backup stored in homologous chromosomes or sister chromatids with the DNA doubles up is broken along both strands. It is essential for sustaining DNA replication, healing double‐strand breaks (DSBs), and facilitating meiosis in somatic cells [113]. The MRE‐11‐RAD50‐NBS1 (MRN) complex serves as a vigilant guardian, recognizing the presence of DSBs within the DNA landscape. Alongside this vigilant sentinel, both ATR and ATM join forces with p53 to sense these potentially catastrophic disruptions [114]. Upon detecting a DSB, the MRN complex swings into action, activating the ATM kinase, which acts as the initiator of the mechanism for DNA damage response. This cascade of events sets the stage for repair mechanisms to kick into gear [115]. BRCA1, another key player in this intricate dance, is drawn to the damaged DNA ends. It displaces p53 and catalyzes a crucial step: the stimulation of 5′ to 3′ end resection through the action of exonucleases. This process generates single‐strand DNA (ssDNA) overhangs, laying the groundwork for the repair process to begin in earnest [116]. The exposed ssDNA, now vulnerable to the elements, finds protection in the form of DNA replication protein A (RPA). This coating of RPA not only shields the ssDNA but also activates the ATR response, a vital trigger for HR repair [117]. Concurrently, the ATR‐Chk1 DNA damage checkpoint is engaged, halting the cell cycle to provide a breathing space for repair machinery to operate effectively and safeguarding stalled replication forks from further harm. With the stage set and the actors in position, RAD51 steps into the spotlight, aided by the supporting roles of BRCA2 and PALB2 (Figure 5) [118]. Together, they orchestrate a meticulous ballet of homology sequence searching and strand invasion, laying the groundwork for precise repair. As the repair process unfolds, DSBs are methodically restored through a choreographed DNA synthesis sequence, ligation, and Holliday junction repair [119]. This intricate dance of molecular machinery ensures that because the genome's integrity is maintained, the blueprint of life itself from the ravages of DNA damage [120].

Triptolide downregulated ATM expression and impaired the DNA damage response to double‐strand breaks. Furthermore, the chemosensitizing effect of Triptolide on Doxorubicin was reduced upon ATM overexpression in breast cancer cells [121]. Resveratrol increased the sensitivity of breast cancer cells to cisplatin, particularly in cisplatin‐resistant MCF7 cells. It achieved this by reducing the expression of several critical components involved in the HR repair pathway, effectively resensitizing the cells [122]. Curcumin enhances the apoptotic effects of cisplatin in cisplatin‐resistant lung adenocarcinoma cells by inhibiting FANCD2 monoubiquitination, thereby preventing the activation of the Fanconi anemia/BRCA pathway, which is crucial for DNA repair through HR [123]. Genistein suppressed both HR repair pathways in glioblastoma and sarcoma cells following damage induced by carbon ion radiation. This effect is likely due to genistein's ability to inhibit the phosphorylation of DNA‐PKcs and KU80, which in turn delays the formation of RAD51 foci [124]. Triptolide was found to reduce the levels of PARP1, XRCC1, and RAD51 proteins in triple‐negative breast cancer cells, impacting the repair of the HR pathway [98]. Cantharidin, a terpenoid compound secreted by various blister beetle species, has been shown to enhance the sensitivity of pancreatic cancer cells to ionizing radiation. It does so by increasing levels of phosphorylated H2AX and modulating the expression of several proteins, including UBE2T, RPA1, GTF2H5, LIG1, POLD3, RMI2, XRCC1, PRKDC, FANCI, FAAP100, RAD50, RAD51D, RAD51B, and DMC1, which are crucial for repair via the HR pathway [125]. DNA damage induced by the carcinogen NKKOAc triggers ATR activation, leading to the phosphorylation of γ‐H2AX and the activation of Chk1. This initiates the DNA damage response through various effector proteins, including p53 and BRCA1, which facilitate DNA repair via single‐strand break repair and HR pathways [49].

### 5.5 Nonhomologous End Joining Pathway

The NHEJ pathway, which is primarily active throughout the cell cycle's G0 and G1 phases and is dependent on DNA‐dependent protein kinase (DNA‐PK) enzyme complex, repairs the majority of DSBs [126]. Among the primary protein complexes in “NHEJ are the Ku subunits (Ku70/Ku80), DNA‐PKcs, DNA ligase IV (Lig4), its cofactor, the X‐ray cross complementation group 4 protein (XRCC4), and the nuclease artemis” [127]. The ring‐shaped Ku70 and Ku80 subunits initiate the process of NHEJ by identifying and binding the damaged DNA [128]. It interacts with DNA and Ku to produce this recruit monomeric DNA‐PKcs. DNA‐PKcs form the heterodimer DNA‐PK together with the Ku subunits. Subsequently, the DNA‐PKcs form a synaptic complex by dimerizing and binding across the DNA termini [129]. DNA‐PKcs can be recruited by DNA‐PKcs serving as an anchor damaged DNA ends and to assist the Ku heterodimers relocation into the DNA helix [127]. Additionally, it is suggested that DNA‐PKcs align DNA breaks and shield DNA from exonucleolytic destruction. DNA‐PKcs functions as a scaffolding protein in this manner, facilitating the removal of damaged DNA and the arrival of repair proteins. Other proteins like Ku70/80, XRCC4, Lig4, and Artemis that mediate NHEJ are actively altered and phosphorylated by DNA binding. Additionally, it raises DNA‐PKcs' kinase activity [130]. Lig4 and XRCC4 mediate the ligation of DNA ends. The Lig4‐XRCC4 complex has been found to bind to another factor, XRCC4‐like factor (XLF), which is required for effective ligation by NHEJ [131]. Moreover, active DNA‐PKcs phosphorylate Ser139 at histone variant H2AX is a well‐known hallmark for DSBs. This marker coordinates the communication cascades required for successful repair and draws repair parts to the damaged area [132]. Its binding site has an impact on DNA‐PK activity and activation. While the 3′ end of the DNA anneals the DNA terminals along the break, the kinase is activated by the DNA's 5′ end [133].

**FIGURE 6:**
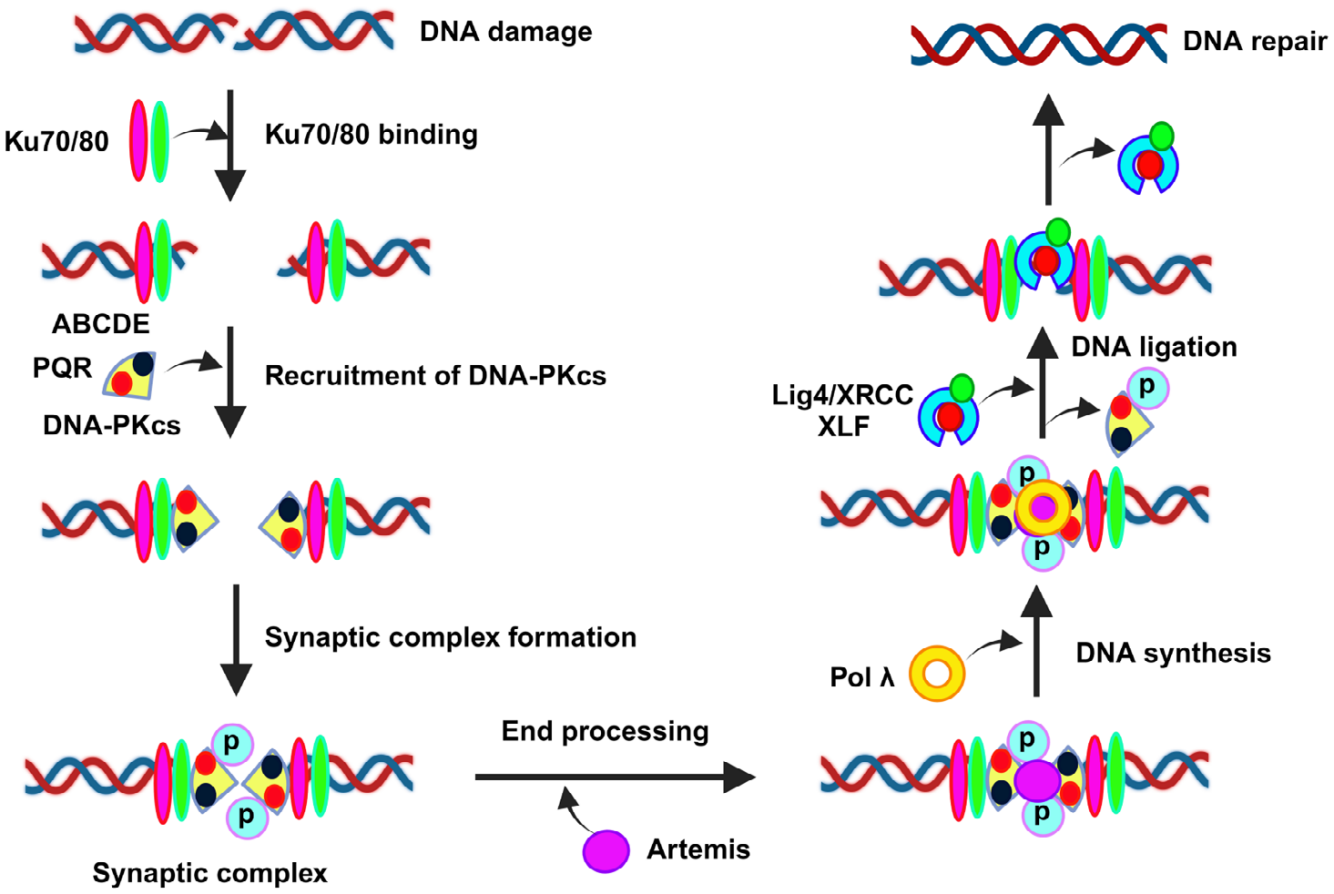
Nonhomologous end joining pathway. Nonhomologous end joining (NHEJ) is a critical repair pathway for double‐strand DNA breaks. The process begins when Ku70/80 binds to the broken DNA ends, recruiting DNA‐PKcs to form a synaptic complex. This complex facilitates the recognition and alignment of the broken DNA ends. DNA polymerase λ is then recruited to fill any gaps, and Artemis processes the DNA ends, a step activated by DNA‐PKcs autophosphorylation. Once further autophosphorylation occurs, DNA‐PKcs dissociates, and XLF collaborates with the Lig4‐XRCC4 complex to finalize DNA ligation, ultimately repairing the break and restoring the DNA structure.

The relationships between Ku80 and DNA‐PKcs are essential for DNA‐PK activity, which are not restricted to structural area of the Ku80 C‐terminus, as demonstrated by mutation studies of Ku subunits and DNA‐PKcs. Furthermore, each structural area of the Ku80 C‐terminus needs to be present for the kinase activity to be initiated. It was also found that DNA‐PK activation and the Ku80/DNA‐PKcs interaction are influenced by the structural characteristics of the substrate, including DNA extensions, length, direction, and sequence of extensions (Figure 6) [134]. The activity of DNA‐PK is necessary for its function in NHEJ [135]. Even though many target proteins for DNA‐PK have been discovered and it is currently believed that effective NHEJ does not require DNA‐PK to phosphorylate these proteins [136]. According to a recent study, gap‐filling DNA synthesis during NHEJ is facilitated by DNA polymerase λ and is mediated by phosphorylation of DNA‐PK [137]. The DNA‐PK catalytic subunit is the primary target location for phosphorylation of DNA‐PK [138]. Enzyme inactivation, complex dissociation, and end processing control all depend on DNA‐PK autophosphorylation. DNA end access is controlled by the autophosphorylation of two residue clusters, ABCDE and PQR, in preparation for ligation and processing [139]. According to mutational research, phosphorylation inside the PQR cluster inhibits end processing, while in ABCDE, phosphorylation exposes the ends to be processed [140]. These phosphorylation events suggest a method via which DNA‐PK shields the ends of the DNA and permits processing only when necessary. Furthermore, kinase activity is reduced because of DNA‐PKcs autophosphorylation, which causes DNA‐PKcs to detach from the complex of Ku‐DNA. It appears that ABCDE and PQR sections are necessary for the DNA to separate from PKcs [141]. Although NHEJ is the primary mechanism in the S/G2 phase of the cell cycle, DNA repair in G1, NHEJ, and HR are directly competing is shown by the production of NHEJ components at every stage of the cell cycle [142]. This implies that even in the case that recruitment of DNA‐PK to the DSB beforehand, there may be a mechanism that promotes HR. One theory is that autophosphorylation of DNA‐PK might facilitate the regulation of HR and NHEJ. End processing is unaltered, and DNA‐PKcs autophosphorylation at T, J, and K boosts HR and prevents certain DSBs from NHEJ [143]. Consequently, it is unknown what mechanism facilitates the JK cluster autophosphorylation. NHEJ may be favored over HR due to Ku recruits to DSB sites more quickly than RAD51 does, and DNA‐PKcs/Ku is available throughout the cell cycle [144]. It's also feasible that HR won't be enabled until NHEJ fails, with NHEJ serving as the standard mechanism for DSB repair [145].

Curcumin helps prevent DNA damage in lymphocytes of individuals chronically exposed to arsenic while enhancing their repair capabilities. It achieves this by promoting the expression of proteins involved in the NHEJ pathway, thereby aiding in the prevention of carcinogenesis [97]. Genistein was shown to block the NHEJ pathway in glioblastoma and sarcoma cells following carbon ion radiation‐induced damage. This effect is likely due to genistein's inhibition of DNA‐PKcs and KU80 phosphorylation, as well as its ability to delay RAD51 foci formation [124]. In cancer cells, curcumin disrupts the NHEJ pathway by inhibiting the acetyltransferase activity of CBP on histones at double‐strand breaks. This prevents the recruitment of KU70/KU80 proteins and p300 to the BRCA1 promoter, leading to the downregulation of BRCA1 expression. Additionally, curcumin inhibits ATR kinase activity, resulting in cell cycle arrest at the G2 phase [146]. Cantharidin, a terpenoid compound secreted by various blister beetle species, has been found to enhance the sensitivity of pancreatic cancer cells to ionizing radiation. It does so by increasing phosphorylated H2AX levels and influencing the expression of key proteins such as UBE2T, RPA1, GTF2H5, LIG1, POLD3, RMI2, XRCC1, PRKDC, FANCI, FAAP100, RAD50, RAD51D, RAD51B, and DMC1. These proteins play crucial roles in DNA repair through the NHEJ pathway [125]. Garcinol, a polyisoprenylated benzophenone derived from the fruit rind of *Garcinia indica* , enhances the sensitivity of cervical cancer cells to ionizing radiation. It does so by inhibiting the NHEJ pathway, particularly by preventing chromatin remodeling, with a focus on histone acetylation [147]. Withanolide D, a compound derived from *Withania somnifera* , has been found to enhance the radiosensitivity of various cancer cell lines by inhibiting DNA repair through the NHEJ pathway [148].

## 6 Dietary Antioxidants as Therapeutic Agents for Genomic Instability

Dietary antioxidants have drawn a great deal of focus during the last 20 years for their potential use in the treatment of conditions like obesity, cancer, Parkinson's disease, inflammatory bowel disease, and cardiovascular illnesses [149]. Numerous investigations have previously been carried out to show the part flavonoids play in various pathological situations, mostly through their pharmacological and biochemical properties, including their prooxidant and antioxidant capacities, which are correlated with their structure–activity connection [150]. Antioxidants can help protect normal cells, but they may be ineffective for cancer cells, which usually have altered proliferation potential [151, 152]. Dietary antioxidants can scavenge radicals in a variety of ways. They can do this by either boosting natural antioxidant enzymes including CAT, GPX, and SOD, or by inhibiting enzymes or chelated trace metals that generate an excessive amount of ROS. Conversely, flavonoids' prooxidant properties can help prevent cancer by preventing tumor cells from proliferating through the triggering of apoptosis. According to recent structure–activity relationship studies, flavonoids exhibit strong antiproliferative properties, particularly when they possess dihydroxy groups at positions 3′ and 4′, a double bond between C2 and C3, and a carbonyl group at C4. However, flavonoids with a functional chemical component in the C7–C8 region had negligible to no antiproliferative effects [153]. Flavonoids regulate transcription factors involved in DNA damage response, inflammation, cell cycle progression, and apoptosis, including NF‐κB, Nrf2, and AP‐1, all of which play key roles in tumor development. Dietary antioxidants such as vitamins, flavonoids, polyphenols, phenolic acids, and alkaloids have shown promising results in both preclinical and clinical studies [30].

Both men and women who consume flavonoids, including flavanones and anthocyanins, have a lower risk of cardiovascular diseases (CVDs). Research on 43,880 healthy males who regularly consume more anthocyanins revealed a decreased risk of ischemic stroke during a 24‐year period [94]. Numerous studies have demonstrated that anthocyanins positively affect LDL‐cholesterol levels by modulating cholesterol metabolism and influencing key pathways involved in lipid regulation [154]. Through a reduction in adult hypercholesterolemia, anthocyanins demonstrated anti‐inflammatory effects in a randomized controlled experiment. This result was in keeping with earlier findings, since anthocyanin consumption dramatically lowered levels of HDL and LDL cholesterol while inhibiting HepG2 cell line‐derived IL‐6 and IL‐1β [155]. Furthermore, dietary flavonoid consumption directly influences microbiota because it is known that ingested flavonoids remain inactive in the small intestine and make their way to the colon. In this environment, the microbes create a variety of enzymes that help with the fermentation and hydrolysis of flavonoids. Microbes can help convert flavonoids into aromatic catabolites and phenolic acids through the processes of oxidation, demethylation, and breakdown [156]. According to recent research, there is an inverse relationship between obesity and inflammation and a diet high in flavonoids. Because it raises adiposity and fatty acid metabolism, gut microbiota plays a major role in mediating this. Additionally, intestinal microorganisms change flavonoids into monomers that may be more readily absorbed in the intestine by glucosidation, dihydroxylation, and decarboxylation [157]. Furthermore, in mice fed a high‐fat diet, flavonoids extracted from mulberry leaves improve lipid dysmetabolism. Bacteroidetes and other gut bacteria contributed to the increased synthesis of acetic acid, which helped to restore lipid metabolism [158].

## 7 PDT‐Mediated DNA Repair

Photodynamic therapy is equally effective in both MMR‐deficient and MMR‐proficient cells, with no observed loss of MMR function after repeated treatments. MMR‐deficient cells also show similar m‐THPC uptake and distribution, supporting PDT as a potential strategy to overcome resistance linked to MMR deficiency [159]. Studies have revealed that, while PDT damages DNA, depending on where the PS is located, DNA repair mechanisms fix the damage swiftly. DNA repair has been shown to occur within 4 h after PDT, and the issue rests with the repair. There is no guarantee that the repaired DNA will be free of mutations. Some forms of PS, such as haematoporphyrin derivatives, have been related to blocking DNA repair enzymes, thus directly impacting DNA repair [160]. Recent investigations have demonstrated that p53 inhibits photofrin‐mediated PDT [161]. P53 interacts with the promoter of alkylation repair homolog 2 (ALKBH2). The ALKBH2 protein is important for repairing alkylated DNA by oxidative methylation [161]. The PCNA is a reliable biomarker of cell proliferation and DNA repair. PCNA is a critical factor in DNA metabolism, functioning primarily as a processivity factor for POLD during replication. It also plays key roles in DNA repair and recombination by coordinating the recruitment of various proteins involved in these pathways [162]. Additionally, PCNA plays a similar role in DNA repair by facilitating the activity of POLB and recruiting other repair proteins. We will revise the manuscript to clarify these points and eliminate any ambiguity [163]. PDT has been demonstrated to raise the level of PCNA in cells, causing DNA damage repair and avoiding necrosis. New research indicates that combining PDT with β‐glucans improves its effectiveness. β‐glucans are polysaccharides present in the cell walls of yeast, fungus, and pathogenic bacteria. β‐glucans inhibit tumor growth and proliferation by activating complement receptor 3 (CR3), leading to necrosis. Cells treated with β‐glucans have lower levels of PCNA, indicating less DNA repair [164].

## 8 Optimizing PDT With Dietary Antioxidants

Photodynamic therapy is a novel anticancer approach that destroys tumors by generating ROS and singlet oxygen through Type I and Type II photochemical reactions [2]. The effectiveness of PDT largely relies on intracellular ROS levels, but cellular antioxidant defenses can counteract ROS‐induced cell death [165]. PDT causes DNA damage mainly by generating ROS, which result in oxidative lesions like single‐ and double‐strand breaks, base oxidation, and DNA‐protein crosslinks. If not properly repaired, these alterations can compromise genome integrity and lead to cell death [166]. PDT is primarily used for treating superficial and early‐stage cancers, including skin, esophageal, bladder, and nonsmall cell lung cancers. In advanced or recurrent cases, it can also serve as a palliative approach to alleviate symptoms and decrease tumor mass [167]. PDT enhances treatment selectivity by using PSs that preferentially accumulate in tumor cells. Upon activation by localized light, these PSs generate ROS that selectively kill cancer cells while minimizing damage to surrounding healthy tissue [2]. Dietary antioxidants do not directly enhance the cytotoxic effects of PDT; certain natural compounds with antioxidant properties may improve its targeting efficiency by modulating the tumor microenvironment, enhancing PS accumulation in tumor cells, or reducing off‐target oxidative stress [168]. FDA‐approved antioxidants like vitamin C, vitamin E, and selenium may enhance PDT efficacy by reducing oxidative damage, supporting repair mechanisms, and improving therapeutic response [169]. Selective protection of healthy tissues during PDT using dietary antioxidants reduces oxidative damage caused by ROS, allowing higher or repeated treatment doses with fewer side effects. This approach widens the therapeutic window, enhancing tumor targeting while preserving normal tissue function and improving patient outcomes [170, 171]. The long‐term impact of dietary antioxidants often varies with their origin. Whole foods typically offer greater benefits because of naturally occurring synergistic compounds, whereas supplements deliver higher concentrations but may not include the complete spectrum of nutrients found in food [172]. The success of PDT combined with dietary antioxidants may vary depending on age, cancer type, and tumor location. Aging can reduce the body's natural antioxidant defenses and DNA repair efficiency, while different tumors or anatomical sites may show varied responses due to differences in oxygen availability, PS absorption, and antioxidant presence [173]. Primary endpoints for assessing the benefits of dietary antioxidants include lower levels of oxidative stress markers, enhanced DNA repair capacity, and better treatment outcomes. Other factors to consider are tumor shrinkage, improvements in patients' quality of life, and reduced damage to healthy tissues during therapy [87]. Secondary endpoints for PDT combined with antioxidant therapy might include tracking side effects, immune response changes, survival rates, and progression‐free survival. Additional factors could involve assessing tumor recurrence, quality of life improvements, inflammation or oxidative stress biomarkers, and long‐term effects on tissue repair and regeneration [168]. MutT Homolog 1 (MTH1) is a promising therapeutic target in cancer due to its role in protecting cells from oxidative damage by sanitizing the oxidized nucleotide pool. MTH1 inhibitors have been developed to selectively induce oxidative stress in cancer cells, though their effectiveness varies. Its structure and function suggest broader roles in cancer progression and oxidative stress regulation in various diseases [174].

## 9 Antioxidants in PDT: Clinical Trials

A detailed summary of clinical trials from March 2013 to March 2023 was compiled using the keywords “cancer” and “PDT” from ClinicalTrials.gov [175]. PDT was the first FDA‐approved drug/device combination, and several PSs have since been commercialized or evaluated in clinical trials [176]. Edaravone was the first dietary antioxidant drug approved in Japan and South Korea for the treatment of amyotrophic lateral sclerosis, and later received FDA approval as well [177]. New strategies are being developed to enhance the delivery of PSs to tumor tissues, improve targeting specificity, and boost therapeutic efficiency [178]. Several dietary antioxidants have been investigated as PSs in preclinical cancer studies, but none are currently FDA‐approved for clinical application. Continued clinical trials and proof‐of‐concept research are needed to support their potential in enhancing PDT.

## 10 Conclusions and Future Directions

In conclusion, the integrity of PDT with dietary antioxidants is a potential technique for boosting genomic stability and DNA damage repair mechanisms. The oxidative stress caused by PDT can cause severe DNA damage; however, the concomitant use of dietary antioxidants appears to attenuate these negative effects by strengthening the body's innate antioxidant defenses. Dietary antioxidants may improve cellular resilience to oxidative damage and contribute to healthier outcomes after PDT by promoting various DNA repair pathways such as BER, NER, MMR, HR, and NHEJ. Future studies should elucidate the precise biochemical pathways by which dietary antioxidants protect DNA integrity during PDT. Investigating the synergistic interactions of antioxidants and PDT photosensitizers may identify the best combinations for maximizing therapeutic efficacy while avoiding oxidative damage to healthy tissues. Furthermore, clinical trials are required to evaluate the combination approach's real‐world applicability, finding the most effective types and quantities of dietary antioxidants. Understanding the long‐term consequences of this method for genetic stability, cancer prevention, and overall health is crucial. By researching further into these relationships, researchers can pave the road for novel therapeutic approaches that not only improve cancer treatment outcomes but also boost overall health and longevity.

## Author Contributions

**J. P. Jose Merlin:** writing – original draft. **Sheeja S. Rajan:** writing – original draft. **Heidi Abrahamse:** supervision, writing – review and editing.

## Conflicts of Interest

The authors declare no conflicts of interest.

## Data Availability

The information provided in this evaluation is not openly accessible.
